# High Temperature Promoted the Accumulation of Citrus Yellow Mosaic Virus in *Citrus sinensis* via Weakening the Immune Function of the *Cs*WRKY76‐ *Cs*PR4A Modules

**DOI:** 10.1111/mpp.70161

**Published:** 2025-10-05

**Authors:** Xu‐Bin Tian, Xinliang Wang, Yayu Li, Jiaxin Li, Jinhuan Zhou, Zhen Song

**Affiliations:** ^1^ Citrus Research Institute, Southwest University/National Citrus Engineering Research Center Chongqing China; ^2^ Southwest University, Integrative Science Center of Germplasm Creation in Western China Chongqing China

**Keywords:** *Citrus yellow mosaic virus*, *CsPR4A*, *CsWRKY76*, high‐temperature

## Abstract

Rising global temperatures exacerbate the severity of crop diseases, threatening global agricultural production. Citrus yellow mosaic virus (CYMV) is one of the pathogens that seriously threaten citrus production, the world's largest fruit industry. However, the molecular mechanisms underlying CYMV–citrus interactions at high temperatures remain poorly understood. Over a 1‐year observation period, this study found elevated temperatures increased CYMV accumulation in Madam Vinous sweet orange. Controlled experiments comparing 25°C and 37°C conditions further validated this phenomenon, with significantly higher viral titres observed under high‐temperature treatments (37°C). Subsequent transcriptomic analysis revealed that the transcription factor *CsWRKY76* and the pathogenesis‐related gene *CsPR4A* were significantly downregulated in sweet orange infected with CYMV at 37°C. *Cs*WRKY76 could directly bind to the *CsPR4A* promoter, thereby positively regulating *CsPR4A* transcription. Overexpression of *CsWRKY76* or *CsPR4A* in transgenic citrus hairy roots significantly suppressed CYMV accumulation, while RNAi‐mediated silencing of either gene promoted viral accumulation, indicating that both genes were positive regulators of citrus immunity. Overexpression of *CsWRKY76* increased hydrogen peroxide content in transgenic citrus hairy roots while upregulating *CsPAL2* and *CsCOMT1* (involved in phenylpropanoid metabolism). This study elucidates the molecular mechanism by which high temperature suppresses the immune function of the *Cs*WRKY76‐*Cs*PR4A modules and thereby promotes the accumulation of CYMV. Our results provide a theoretical basis for developing high‐temperature‐resistant disease control strategies in citrus.

## Graphical abstract

The content of citrus yellow mosaic virus (CYMV) increased with rising temperature. This study has undertaken additional research on the molecular regulatory mechanism that *WRKY76* and *PR4A* are important regulatory factors that enhance citrus resistance to CYMV.

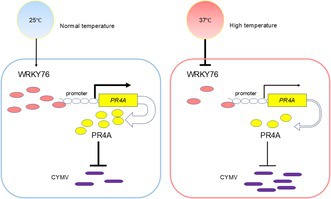

## 1 Introduction

In recent years, extreme weather conditions, characterised by elevated temperatures, had a detrimental effect on the growth and development of plants, including root growth, flowering, pollination, fruiting, filling and seed ripening (Lippmann et al. 2019). This ultimately reduces crop yield. Simultaneously, elevated environmental temperatures exacerbate the spread of plant viruses by accelerating viral replication and vector activity, further compromising agricultural productivity (Tsai et al. 2022). In *Brassica campestris*, the systemic infection progression of turnip mosaic virus (TuMV) accelerates linearly as temperatures rise between 13°C and 23°C (Chung et al. 2015). The TuMV coat protein (CP) accumulation is significantly higher at 23°C–28°C (Chung et al. 2015). However, beyond a virus's optimal thermal threshold, this progression reverses (Tsai et al. 2022). For instance, the systemic infection rate of potato virus Y (PVY) increases with rising temperatures within the range of 20°C–28°C. However, once the temperature exceeds 35°C, PVY systemic infection ceases (Choi et al. 2017). TuMV also exhibits a similar phenomenon: when temperatures reach 33°C, symptoms in TuMV‐infected *B. campestris* are delayed, and CP accumulation is reduced (Chung et al. 2015).

The ambient thermal conditions and plant–virus interactions form a bidirectional relationship, influencing both viral infection and plant defence mechanisms. Plants engage effector‐triggered immunity (ETI) signalling at relatively low temperatures (10°C–23°C), while they transition to pattern‐triggered immunity (PTI) signalling at moderately elevated temperatures (23°C–32°C) (Cheng et al. 2013). TCP transcription factors (TEOSINTE BRANCHED1, CYCLOIDEA, AND PROLIFERATING CELL FACTOR) induced by elevated temperature physically interact with ZRKs (HOPZ‐ETI‐DEFICIENT 1‐related kinases). This interaction inhibits the plant immune response by suppressing the transcription of SNC1 (SUPPRESSOR OF NPR1‐1, CONSTITUTIVE 1) (Wang et al. 2019). When plants are subjected to both high temperatures and pathogens simultaneously, they prioritise enhancing heat tolerance over pathogen resistance (Saijo and Loo 2020). Therefore, plants in high‐temperature environments exhibit a temporary reduction in immune responses, such as membrane fluidisation, production of reactive oxygen species (ROS) and accumulation of secondary metabolites (Mathur et al. 2014). This renders plants in high‐temperature environments more susceptible to pathogen infection (Desaint et al. 2021; Saijo and Loo 2020).

Citrus yellow mosaic virus (CYMV) is a double‐stranded DNA virus, belonging to the genus *Badnavirus* in the *Caulimoviridae* family (Anthony Johnson et al. 2012). CYMV causes a serious disease in citrus crops, characterised by symptoms such as leaf yellowing, deformity or curling. In severe cases, necrotic spots may also develop (Anthony Johnson et al. 2017; Ghosh et al. 2014). In southern India, CYMV has become second only to citrus tristeza virus in importance (Anthony Johnson et al. 2017). Rapid diagnostic methods for CYMV detection in quarantine systems have been established to counter CYMV's spread via global trade (Kumar et al. 2018; Motghare et al. 2018). However, no prior studies have investigated whether rising temperatures exacerbate CYMV infection of citrus plants, a question of urgent relevance under climate change scenarios.

The present study revealed that elevated temperatures promoted CYMV accumulation in citrus plants. Using transcriptome sequencing, we offer information about the regulatory mechanisms underlying citrus gene expression responses to CYMV under high‐temperature conditions. Two host genes, namely *CsWRKY76* and *CsPR4A*, formed a *Cs*WRKY76‐*Cs*PR4A regulatory module that suppressed CYMV accumulation. These genes could be potential genetic breeding targets that could contribute to the prevention and management of CYMV.

## 2 Results

### 2.1 High‐Temperature Promoted the Accumulation of CYMV

**FIGURE 1:**
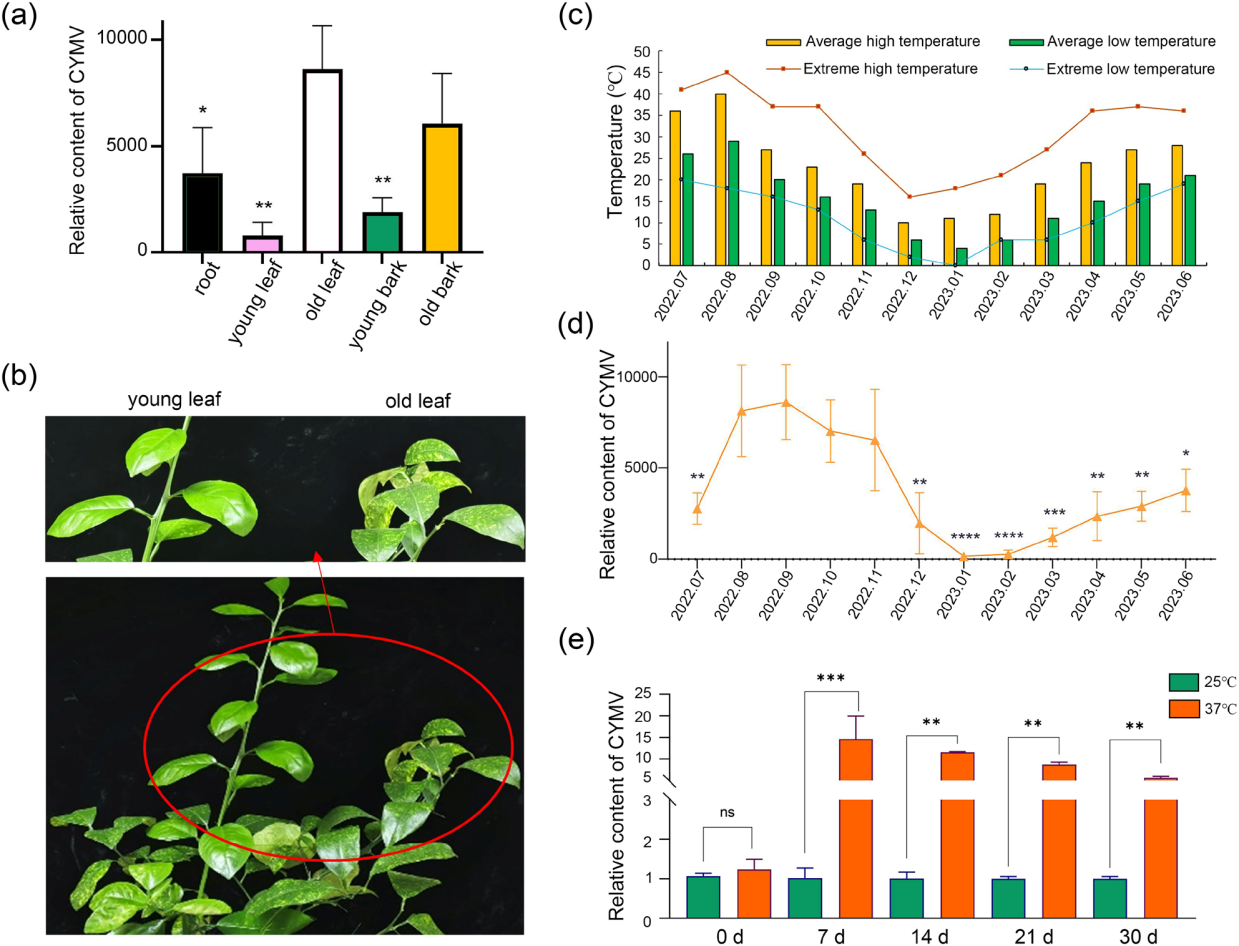
High temperature promoted the accumulation of citrus yellow mosaic virus (CYMV) in Madam Vinous sweet orange. (a) The relative content of CYMV in different citrus tissues. (b) Foliar symptoms were observed on the old leaves of 2‐year‐old Madam Vinous sweet oranges following the inoculation with CYMV. (c) Temperature records for Chongqing from July 2022 to June 2023. (d) CYMV content from July 2022 to June 2023. (e) Viral content in Madam Vinous sweet orange plants infected with CYMV at Days 0, 7, 14, 21 and 30 in two temperature regimes: 25°C and 37°C. Asterisks indicate significant differences by two‐way ANOVA followed by Dunnett's multiple comparisons test (ns *p* ≥ 0.05, *p < 0.05, **p < 0.01, ***p < 0.001, ****p < 0.0001).

The sweet orange cv. Madam Vinous was selected for investigation into the distribution of CYMV within diverse citrus tissues. The highest viral content was detected in the old leaf tissue, followed by the old bark, roots and young bark. The lowest viral content was observed in the young leaf (Figure 1a). Foliar symptoms were consistently observed on the old leaves of sweet oranges 2 years after CYMV inoculation, but not on the young leaves of new shoots (Figure 1b).

The relative CYMV content was measured in old leaves of sweet orange during the time period from 10 July 2022 to 10 June 2023, while also recording the ambient temperature (Figure 1c,d). The relative CYMV content in the old leaves of sweet orange exhibited an increasing trend from July to September, reaching a peak in viral content. A significant decrease was observed from October to January, reaching the lowest value in January, and then increasing month by month starting from February of the following year (Figure 1c). The trend of the relative viral content was broadly consistent with the trend of temperature change from July 2022 to June 2023 in the greenhouse of the Citrus Research Institute in Chongqing, China (Figure 1d), which initially indicated that the content of CYMV was closely related to the environmental temperature.

In order to further investigate the relationship between temperature and CYMV, CYMV‐positive sweet oranges with uniform growth were selected for growth at 25°C and 37°C. The CYMV content in old leaves was determined at 0, 7, 14, 21 and 30 dpi (Figure 1e). The CYMV content in sweet orange grown at 37°C was significantly higher than that in plants grown at 25°C (Figure 1e). This finding suggests that high temperature promotes the accumulation of CYMV.

### 2.2 Transcriptome Profiling of Madam Vinous Sweet Oranges Infected With CYMV at 37°C Exhibited a Downregulation of Defence‐Related Genes

**FIGURE 2:**
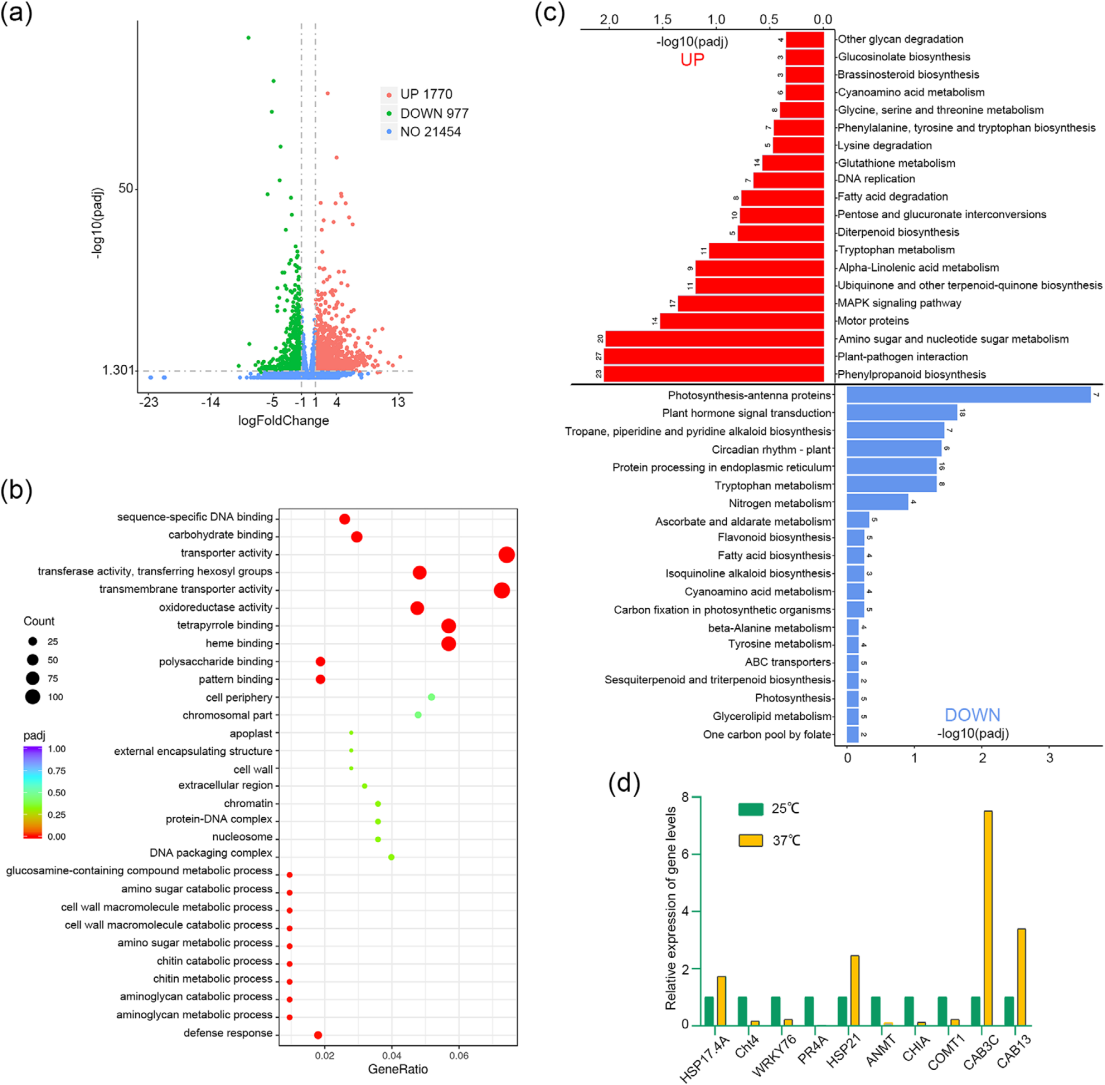
Transcriptome analysis of citrus infected with citrus yellow mosaic virus (CYMV) at 25°C and 37°C. (a) Volcano plot of differentially expressed genes (DEGs). (b) GO enrichment analysis of DEGs. (c) KEGG enrichment analysis of DEGs. (d) The expression levels of *HSP17.4A*, *Cht4*, *WRKY76*, *PR4A*, *HSP21*, *ANMT*, *CHIA*, *COMT1*, *CAB3C* and *CAB13* in citrus grown at 25°C and 37°C after CYMV inoculation were analysed by reverse transcription‐quantitative PCR.

To further investigate the interaction between high temperature, virus and sweet orange host, we performed transcriptomic sequencing on old leaves infected with CYMV grown at 25°C and 37°C after 1 month of exposure. The differentially expressed genes (DEGs) were then used for subsequent analysis. Between the 25°C treatment group and the 37°C treatment group, 2747 DEGs were identified, including 1770 upregulated genes and 977 downregulated genes (Figure 2a and Table S1). The top 30 significant GO terms based on *p*_adj_ values of the significantly up‐ and downregulated DEGs include ‘sequence‐specific DNA binding’, ‘carbohydrate binding’, ‘transporter activity’, ‘transferase activity, transferring hexosyl groups’, ‘transmembrane transporter activity’ and ‘oxidoreductase activity’ (Figure 2b).

DEGs identified during CYMV infection of sweet orange at different temperatures were mapped using KEGG enrichment analysis and were predominantly associated with pathways related to the plant's innate immune defence and stress resistance mechanisms (Figure 2c). A greater number of DEGs associated with plant immunity were overexpressed at 25°C compared with 37°C in sweet oranges infected with CYMV. For example, ‘phenylpropanoid biosynthesis’, ‘plant–pathogen interaction’, ‘amino sugar and nucleotide sugar metabolism’, ‘motor proteins’ and ‘MAPK signalling’ pathways were increased (Figure 2c). In contrast, a subset of DEGs associated with growth and development, including ‘photosynthesis‐antenna proteins,’ ‘plant hormone signal transduction,’ ‘tropane, piperidine and pyridine alkaloid biosynthesis,’ ‘circadian rhythm‐plant,’ ‘protein processing in endoplasmic reticulum and ‘tryptophan metabolism,’ demonstrated a decrease in expression at 25°C (Figure 2c).

Reverse transcription‐quantitative PCR (RT‐qPCR) results confirmed that at 37°C, the genes in sweet oranges that respond to heat stress, such as heat shock proteins *HSP17.4A* and *HSP21*, were upregulated, while the expression of genes involved in disease resistance, such as *Cht4*, *WRKY76*, *PR4A* and *COMT1*, was significantly inhibited (Figure 2d). Based on documented evidence linking *WRKY* and pathogenesis‐related (PR) family genes to plant disease resistance mechanisms (He et al. 2025; Yang et al. 2024), we selected *CsWRKY76* and *CsPR4A* as primary targets for our subsequent research.

### 2.3 CsWRKY76 Positively Regulated the Expression of *CsPR4A* and Both Showed Expression Suppression at 37°C

**FIGURE 3:**
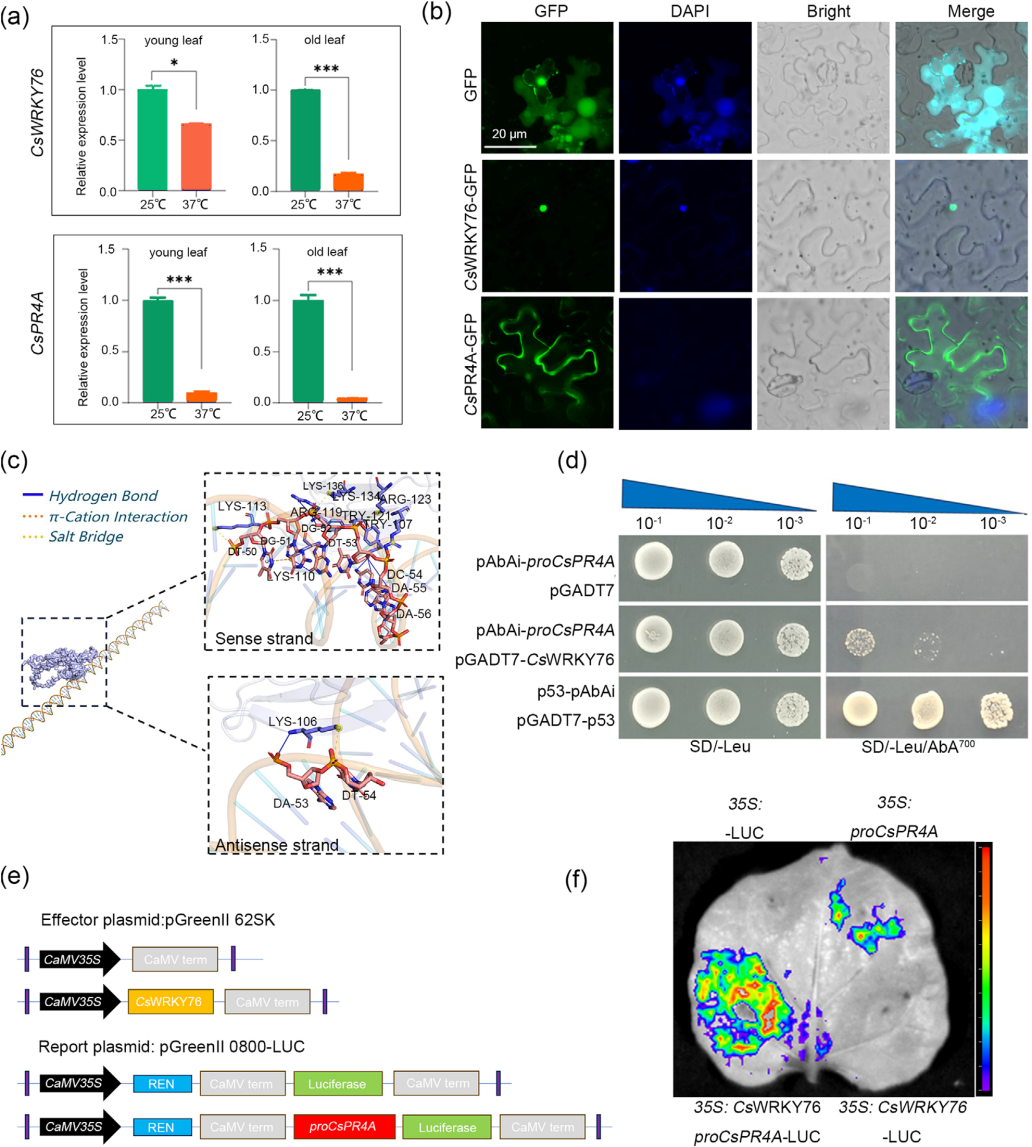
*Cs*WRKY76 induced the expression of *CsPR4A*, and both were suppressed at 37°C (a) The relative expression levels of *CsWRKY76* and *CsPR4A* at 25°C and 37°C. (b) Subcellular localisation of *Cs*WRKY76 and *Cs*PR4A in *Nicotiana benthamiana* leaf epidermal cells following *Agrobacterium*‐mediated transient transformation. The nucleus was stained with DAPI. Bar = 20 μm. (c) The complex structure of *Cs*WRKY76 and *proCsPR4A* promoter DNA fragment was modelled in silico. (d) Yeast one‐hybrid (Y1H) assay indicated *Cs*WRKY76 specifically binds to *proCsPR4A*. Y1HGold [p53‐AbAi] co‐transformed with pGADT7‐p53 was used as a positive control. Y1HGold [pAbAi‐*proCsPR4A*] co‐transformed with pGADT7 plasmid was used as a negative control. The selected transformants were spotted on SD/−Leu and SD/−Leu/+AbA^700^ medium to confirm the interaction. (e) Diagrams of dual‐luciferase activity assays. (f) The dual‐luciferase assay demonstrated that the *CsPR4A* promoter was activated by *Cs*WRKY76 in *N. benthamiana* leaves. The reporter construct was generated by recombining the promoter fragment of *CsPR4A* into pGreenII 0800‐Luc, and *Cs*WRKY76 was cloned into pGreenII 62‐SK to generate the effector construct. The pGreenII 62‐SK‐*Cs*WRKY76 with pGreenII 0800‐LUC*‐CsPR4A* was the test, the pGreenII 62‐SK with pGreenII 0800‐LUC‐*CsPR4A*, pGreenII 62‐SK‐*Cs*WRKY76 with pGreenII 0800‐LUC and pGreenII 62‐SK with pGreenII 0800‐LUC were the controls. The asterisks indicate significant differences using Student's *t* test (*p < 0.05, ***p < 0.001).

RT‐qPCR was used to measure the temperature‐induced changes in *CsWRKY76* and *CsPR4A*. The expression of *CsWRKY76* and *CsPR4A* was significantly inhibited at 37°C (Figure 3a). Inverted fluorescence microscopy showed *Cs*WRKY76‐GFP was located in the nucleus, and *Cs*PR4A‐GFP in the cell membrane, while a fluorescence signal was detected throughout the epidermal cells transformed with empty vector (control) (Figure 3b). The complex structure of *Cs*WRKY76 with the *proCsPR4A* promoter DNA fragment was modelled in silico using Alphafold3, and the interaction details were further examined by PyMOL v. 2.5.4 (Figure 3c). The amino acid sites 104–136 of *Cs*WRKY76 and nucleotides 50–56 of *proCsPR4A* DNA fragments were predicted to interact with each other; the free energy of binding between *Cs*WRKY76 and *proCsPR4A* was −11.6 kcal·mol^−1^, indicating a stable interaction (Figure 3c).

Subsequently, the coding DNA sequence (CDS) of *CsWRKY76* and the promoter fragments of *CsPR4A* were cloned into the pGADT7 activation domain (AD) and pAbAi vectors, respectively. They were then transformed into yeast cells to perform a yeast 1‐hybrid (Y1H) assay. As shown in Figure 3d, only the yeast cells transformed with the vectors pGADT7‐*Cs*WRKY76 and pAbAi containing the promoter region of *CsPR4A*, but not the negative control, survived on selective medium containing aureobasidin A (AbA), indicating that *Cs*WRKY76 interacted with the promoter of *CsPR4A*. The CDS of *Cs*WRKY76 and the promoter fragments of *CsPR4A* were also cloned into the pGreenII 62‐SK and pGreenII 0800‐Luc vectors (Figure 3e), respectively, and then injected into *Nicotiana benthamiana* leaves to perform a dual‐luciferase reporter assay. The results confirmed that co‐expression of *proCsPR4A*‐LUC and 35S:*Cs*WRKY76 significantly increased luciferase activities compared with control combinations in *N. benthamiana* leaves (Figure 3f). These results suggested that *Cs*WRKY76 could bind in planta with *proCsPR4A* and activated the expression of *CsPR4A*.

### 2.4 *CsWRKY76* and *CsPR4A* Silencing Promoted CYMV Accumulation

**FIGURE 4:**
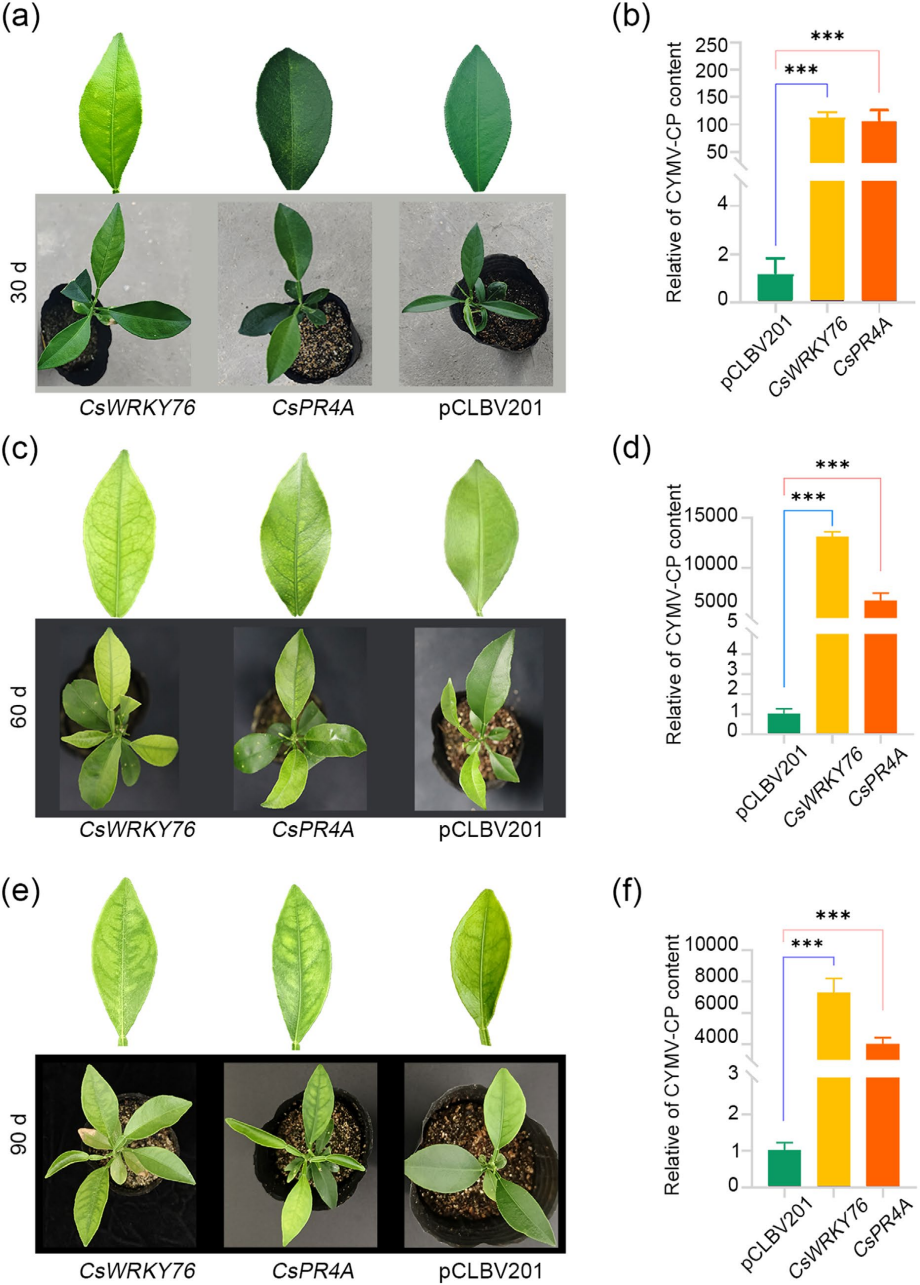
Silencing of *CsWRKY76* and *CsPR4A* promoted the accumulation of citrus yellow mosaic virus (CYMV). (a) The plant phenotypes of virus‐induced gene silencing (VIGS)‐mediated silencing of *CsWRKY76* and *CsPR4A* in ‘jincheng’ sweet oranges 30 days after inoculation with CYMV. (b) The relative quantity of CYMV coat protein gene (*CP*) content in ‘Jincheng’ sweet orange plants with VIGS‐mediated silencing of *CsWRKY76* and *CsPR4A* at 30 days post‐inoculation (dpi). (c) The plant phenotypes of ‘Jincheng’ sweet oranges with VIGS‐mediated silencing of *CsWRKY76* and *CsPR4A* at 60 dpi with CYMV. (d) The relative quantity of CYMV *CP* content in ‘Jincheng’ sweet oranges with VIGS‐mediated silencing of *CsWRKY76* and *CsPR4A* at 60 dpi. (e) The plant phenotypes of ‘Jincheng’ sweet oranges with VIGS‐mediated silencing of *CsWRKY76* and *CsPR4A* at 90 dpi with CYMV. (f) The relative quantity of CYMV‐ *CP* content ‘Jincheng’ sweet oranges with VIGS‐mediated silencing of *CsWRKY76* and *CsPR4A* at 90 dpi. Asterisks indicate significance were examined by one‐way ANOVA multiple comparisons test (***p < 0.001).

To investigate the function of *CsWRKY76* and *CsPR4A* in the defence response against CYMV infection, *CsWRKY76* and *CsPR4A* were silenced in ‘Jincheng’ sweet orange plants using virus‐induced gene silencing (VIGS). The transcript levels of *CsWRKY76* and *CsPR4A* in the silenced plants were quantified by RT‐qPCR assay (Figure S1). The CYMV infectious clone was inoculated into silenced plants by injection, and samples were taken at 30, 60 and 90 days post‐inoculation (dpi), for the RT‐qPCR to detect CYMV. There was a substantial aggravation of the yellowing leaf symptoms in the *CsWRKY76*‐silenced plants in comparison to the control plants at 30 dpi (Figure 4a). The *CsPR4A*‐silenced plants did not manifest any overt chlorosis symptoms (Figure 4a). However, there was a significant increase in CYMV coat protein gene (*CP*) content in both *CsWRKY76*‐ and *CsPR4A*‐silenced plants compared to the control plants (Figure 4b).

By the 60 dpi, all the plants inoculated with CYMV exhibited yellowing leaf symptoms, while the veins of the *CsWRKY76*‐ and *CsPR4A*‐silenced plants were much clearer (Figure 4c). Furthermore, the content of CYMV in the *CsWRKY76*‐silenced plants was more than 10,000 times higher than that of the control group, and the content of CYMV *CP* in the *CsPR4A*‐silenced plants was more than 5000 times higher than that of the control group (Figure 4d). By the 90 dpi, all the plants, with the exception of those that maintained the yellowing leaf phenotype (Figure 4e), still maintained a considerable amount of CYMV *CP* content in comparison to the *CsWRKY76*‐ and *CsPR4A*‐silenced plants and the control group (Figure 4f). However, this showed a downward trend when compared to the content detected at 60 dpi. This finding suggests that the silencing of *CsWRKY76* and *CsPR4A* significantly promoted the accumulation of CYMV.

### 2.5 *CsWRKY76* and *CsPR4A* Positively Regulated the Resistance of Citrus Against CYMV

**FIGURE 5:**
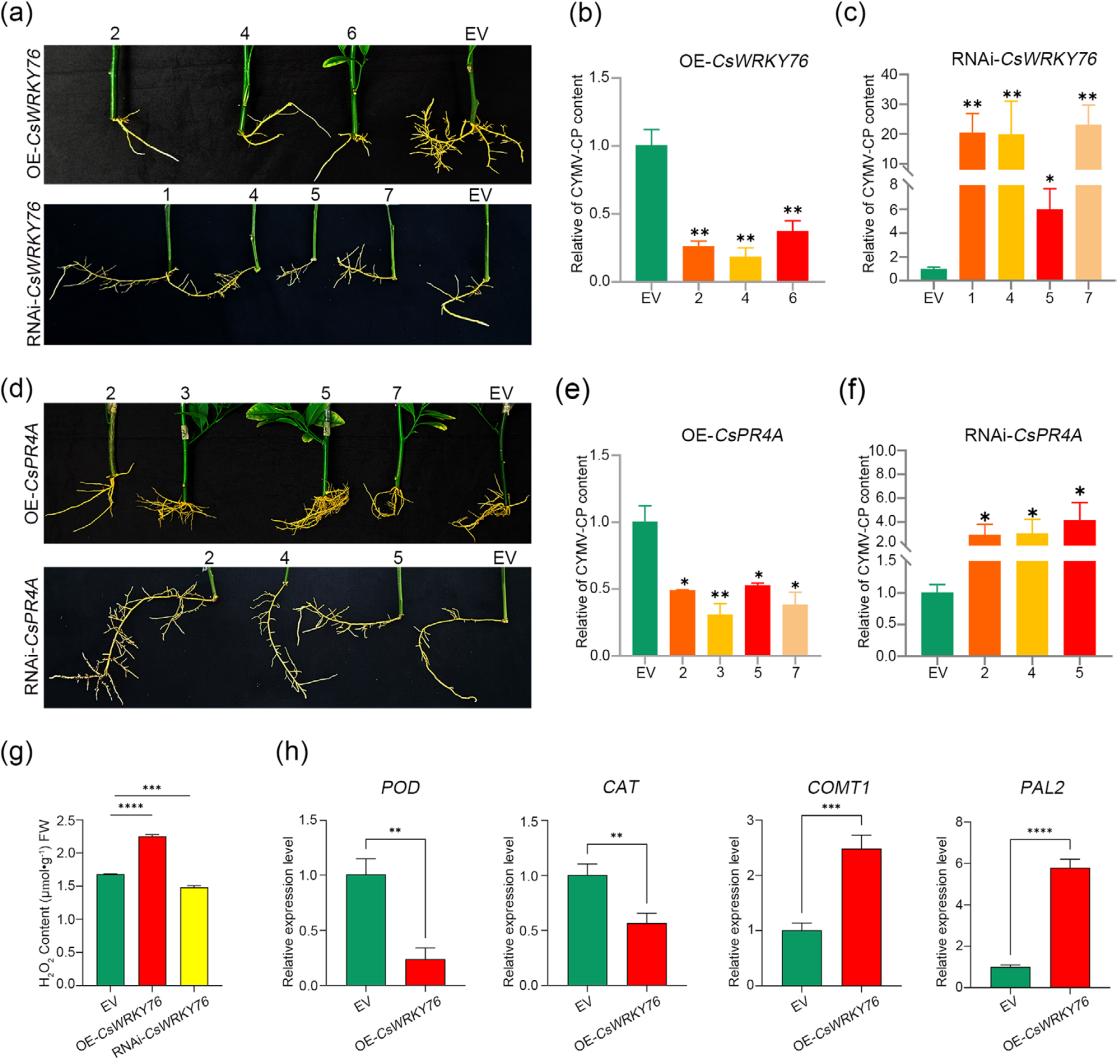
*CsWRKY76* and *CsPR4A* enhanced the resistance of citrus against citrus yellow mosaic virus (CYMV). (a) The OE‐*CsWRKY76* and RNAi‐*CsWRKY76* Madam Vinous sweet orange hairy roots. (b) The relative expression of CYMV coat protein gene (*CP*) in OE‐*CsWRKY76* sweet orange hairy roots. (c) The relative expression of CYMV *CP* in RNAi‐*CsWRKY76* sweet orange hairy roots. (d) The OE‐*CsPR4A* and RNAi‐*CsPR4A* Madam Vinous sweet orange hairy roots. (e) The relative of CYMV *CP* content in OE‐*CsPR4A* sweet orange hairy roots. (f) The relative of CYMV *CP* content in RNAi‐*CsPR4A* sweet orange hairy roots. (g) The content of H_2_O_2_ in OE‐*CsWRKY76* and RNAi‐*CsWRKY76* Madam Vinous sweet orange hairy roots. (h) The relative expression of *POD*, *CAT*, *COMT1* and *PAL2* in OE‐*CsWRKY76* Madam Vinous sweet orange hairy roots. Asterisks indicate significant difference by one‐way ANOVA multiple comparisons test (*p < 0.05, **p < 0.01, ***p < 0.001, ****p < 0.0001).

To further elucidate the role of *CsWRKY76* and *CsPR4A* in CYMV infection, *CsWRKY76* and *CsPR4A* overexpression (OE‐*CsWRKY76* and OE‐*CsPR4A*) and *CsWRKY76* and *CsPR4A* RNA interference (RNAi‐*CsWRKY76* and RNAi‐*CsPR4A*) hairy roots were generated (Figure 5a,b). The transformed hairy roots were examined by a histochemical assay for β‐glucuronidase (GUS) activity and by PCR (Figure S2). The plants that exhibited positive results regarding the levels of gene expression were infected with CYMV through the process of grafting. The roots overexpressing *CsWRKY76* and *CsPR4A* exhibited inhibitory effects on the accumulation of CYMV *CP* (Figure 5c,e). The quantity of CYMV in RNAi‐*CsWRKY76* and RNAi‐*CsPR4A* roots exhibited a significant increase compared to the control group, which was consistent with the results of the VIGS experiment (Figure 5d,f). Hence, both *CsWRKY76* and *CsPR4A* are positive regulators of citrus immunity.

The hydrogen peroxide content (H_2_O_2_) in roots overexpressing *CsWRKY76* was observed to increase, while in RNAi*‐CsWRKY76* roots it showed a reduction (Figure 5g). Through RT‐qPCR experiments, the expression levels of *POD* and *CAT*, which are involved in the degradation of H_2_O_2_, were reduced in the OE‐*CsWRKY76* roots, while the expression levels of *PAL2* and *COMT1*, which are involved in the phenylpropanoid metabolic pathway, were upregulated (Figure 5h). The increase in H_2_O_2_ could effectively inhibit the proliferation of the virus.

## 3 Discussion

Global warming is considered to be one of the most significant environmental challenges currently facing humanity (McMichael et al. 2006). Excessive temperatures exert a detrimental effect on the normal physiological growth of crops (Chaloner et al. 2021). The changes in viral content under different temperature conditions are closely related to the virus's biological characteristics, environmental influences and host factors (Jones and Naidu 2019; Tsai et al. 2022). Some viruses thrive in high‐temperature conditions, thereby increasing viral amount in the host. Temperature affects not only the survival and transmission ability of the virus, but also the host's immunity and physiological metabolism (Tsai et al. 2022).

CYMV has had a profoundly deleterious impact on the citrus industry in some nations (Anthony Johnson et al. 2017). An increase in temperature was conducive to the proliferation of CYMV based on the detailed records of the natural climate in Beibei, Chongqing, China (Figure 1c,d). To control for additional environmental variables (e.g., humidity and light) during seasonal changes, we employed a plant incubator to isolate temperature as the sole variable. The CYMV content in sweet oranges increased nearly 15‐fold within a week at 37°C, remaining elevated for the subsequent month (Figure 1e). Hence, elevated temperature serves as the primary driver promoting CYMV accumulation. Studies on tobacco mosaic virus have demonstrated that a temperature shift from 22°C to 32°C strongly affects the intracellular distribution of the TMV movement protein (MP) and increases the efficiency of cell‐to‐cell viral RNA spread (Boyko et al. 2000). This phenomenon is consistent with the enhanced binding affinity of MP to microtubules across the infection area (Boyko et al. 2000). Microtubules participate in guiding the trafficking of viral replication complexes along the endoplasmic reticulum (ER) and actin network (Niehl et al. 2013). We hypothesise that CYMV may adopt a similar mechanism observed in TMV: under high‐temperature conditions, it increases the binding affinity of MP to microtubules in sweet orange cells. This enhanced binding may improve the CYMV's cell‐to‐cell spread, thereby facilitating its systemic spread and accumulation in citrus plants.

However, compared to analysing how high temperature promoted CYMV accumulation in sweet oranges, we were more concerned about how sweet oranges could resist infection by CYMV under high‐temperature conditions. Consequently, we conducted a comparative analysis of the transcriptome data of sweet oranges infected with CYMV under 25°C and 37°C conditions (Figure 2a). At 37°C, genes linked to plant pathogen resistance, such as MAPK signalling (17 DEGs) and phenylpropanoid biosynthesis (23 DEGs), were downregulated (Figure 2c). MAPKs phosphorylate a variety of substrates, including transcription factors, protein kinases and cytoskeleton‐related proteins, thereby activating the expression of genes related to downstream defence responses (Manna et al. 2023). These indicated that immune signal transmission in sweet oranges infected with CYMV was inhibited at 37°C. The downregulation of phenylpropanoid biosynthesis, which is responsible for synthesising plant stress‐resistance secondary metabolites (e.g., flavonoids and lignin) (Vogt 2010), further indicated that at 37°C sweet oranges exhibited weakened synthesis of stress‐resistance products and were unable to effectively resist CYMV infection. We detected significant upregulation of phenylpropanoid metabolic pathway genes in OE‐*CsWRKY76* transgenic hairy roots (Figure 5h), demonstrating that the phenylpropanoid biosynthesis pathway contributes critically to sweet orange resistance against CYMV infection. In contrast, genes involved in photosynthesis‐antenna proteins and circadian rhythms were downregulated (Figure 2c). There are common signalling components in both light and temperature sensing pathways in plants (Franklin 2009). We speculate that sweet oranges activate the signal pathway responsible for sensing high temperatures, enabling the plants to enhance their tolerance to high‐temperature stress.

Notably, at 37°C, eight DEGs involved in the tryptophan metabolic pathway were upregulated, while 11 DEGs in the same pathway were downregulated (Figure 2c). Tryptophan metabolism generates products such as indole acetic acid (IAA, auxin) and melatonin (Wang et al. 2025). IAA is essential for plant growth and development (Zhao 2012). However, IAA and salicylic acid (SA) exert antagonistic effects in maintaining the dynamic balance between pathogen defence and growth processes (Pokotylo et al. 2022). When a pathogen invades, the accumulation of SA activates the anti‐pathogen response preferentially by inhibiting the IAA signalling pathway (Pokotylo et al. 2022). At 37°C, the SA‐mediated disease‐resistance pathway in sweet orange was clearly disrupted by CYMV, thereby potentially upregulating genes in the tryptophan metabolic pathway (related to auxin synthesis). Another possibility is that the upregulated DEGs at 37°C were primarily responsible for melatonin biosynthesis. Under abiotic stress, melatonin maintains photosynthetic function by elevating chlorophyll levels through enhanced synthesis and reduced degradation (Alyammahi and Gururani 2020). Melatonin also protects photosystems by stabilising photosystem I and preventing photosystem II breakdown, thereby securing an energy supply in harsh environments (Alyammahi and Gururani 2020; Wang et al. 2022, 2025). Miao et al. (2019) showed that exogenous indole promotes the expression of genes involved in SA synthesis and defence responses, enhancing cotton resistance to *Verticillium dahliae*. Therefore, metabolic intermediates in tryptophan metabolism may act as signals to activate SA synthesis. We hypothesise that the DEGs in the tryptophan metabolism pathway that were upregulated at 25°C may promote SA synthesis.

**FIGURE 6:**
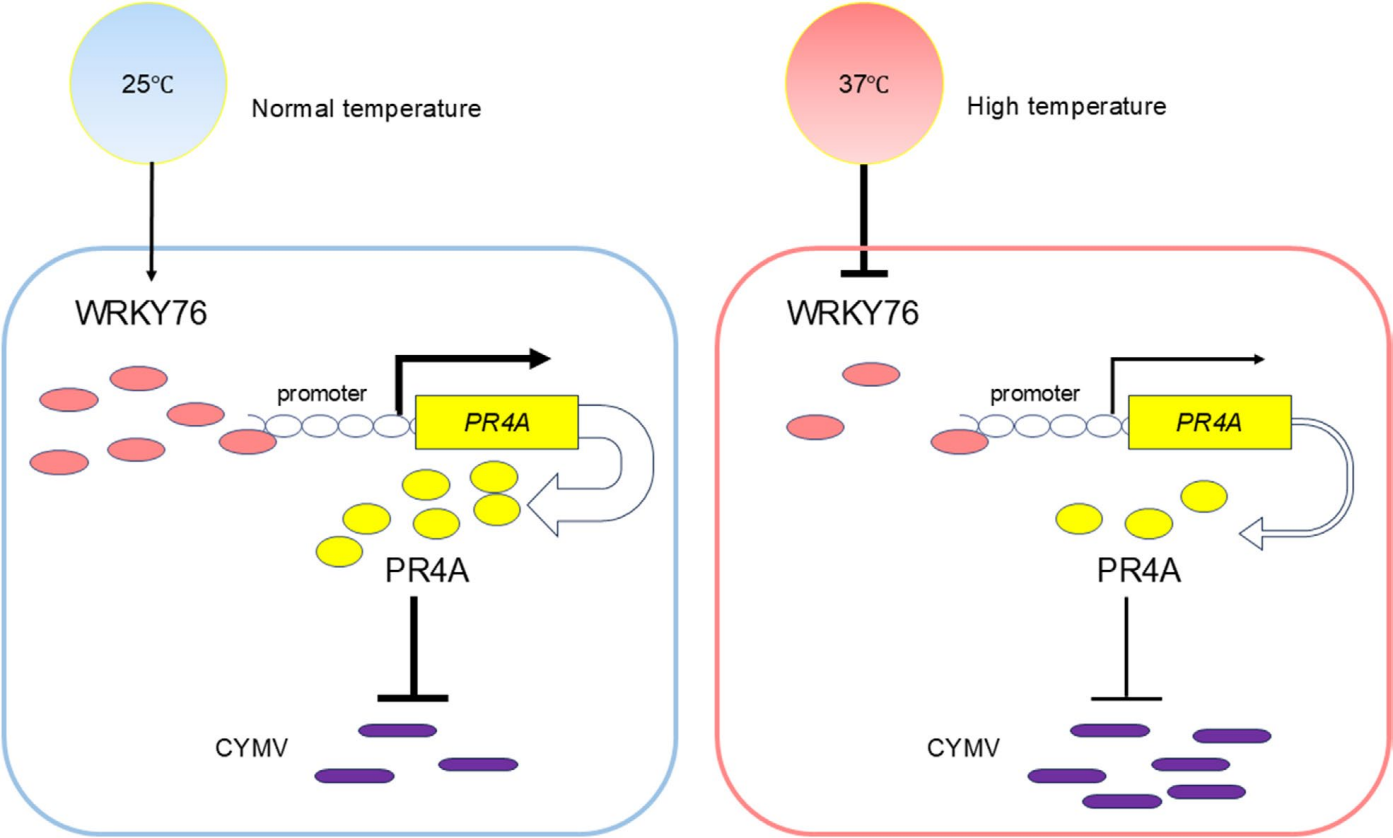
Model of *CsWRKY76*‐mediated defence responses to citrus yellow mosaic virus (CYMV) in citrus plants at 25°C and 37°C. At 25°C (the optimal growth temperature for citrus plants), citrus first activates the expression of *WRKY76*, which then binds to the promoter of *PR4A*. This binding promotes the transcription of *PR4A*, thereby enhancing citrus' resistance to CYMV infection. In contrast, at 37°C (a high‐temperature stress condition), citrus plants suppress *WRKY76* expression in response to heat stress. This suppression inhibits the transcription of *PR4A*), thereby weakening citrus plants' resistance to CYMV. Consequently, CYMV accumulates significantly in citrus plants under hig‐ temperatures.

In response to high temperature stress, plants preferentially upregulate heat shock proteins (HSPs) to maintain protein homeostasis (Mathur et al. 2014). This phenomenon has been observed in both thermosensitive and potato virus Y (PVY)‐susceptible potato varieties, as well as in thermotolerant and PVY‐resistant potato varieties (Makarova et al. 2018). However, in thermotolerant and PVY‐resistant potato varieties, a significant number of PR proteins are induced simultaneously, which may serve as the primary mechanism underlying their resistance to PVY (Makarova et al. 2018). Given the need to mitigate against CYMV infection in thermally stressed environments, elucidating the roles of heat shock proteins (HSPs) became imperative. Transcription factors of the WRKY family are involved in most of the plant growth and development processes and coordinate the response of plants to biotic stresses (Jiang et al. 2017). For instance, *VrWRKY22* from the American grapevine *Vitis rupestris* enhances the resistance of suspension cell lines to *Neofusicoccum parvum* by strengthening the microtubule‐mediated signalling pathway (Tian et al. 2025). The synthesis of SA induced by pathogens primarily relies on the activation of isochorismate synthase (ICS) in the chloroplast. This regulatory mechanism involves multiple transcription factors, such as WRKY28 and WRKY75 (Guo et al. 2017; van Verk 2011), while NRP1 has been identified as the specific receptor for SA. The N‐terminal BTB/POZ domain of NRP1 facilitates its binding to SA, and subsequent studies revealed that NRP1 also binds to TGA transcription factors in a monomeric form, thereby activating the expression of downstream PR genes (Kinkema et al. 2000). In this study, *CsWRKY76* and *CsPR4A* exhibited a substantial decrease in expression during temperature‐mediated viral infection (Figure 3a and Table S1). The VIGS technology and the use of *Agrobacterium*‐mediated transgenic experiments fully demonstrated that *CsWRKY76* and *CsPR4A* had a positive regulatory function on citrus's resistance to CYMV (Figure 4a–f). The molecular mechanism of *Cs*WRKY76 directly binding to the promoter of *CsPR4A* to positively regulate its expression was confirmed through dual‐luciferase and Y1H assays (Figure 3d–f). Hence, *Cs*WRKY76‐*Cs*PR4A modulation plays a critical role in the citrus–CYMV interaction. Furthermore, overexpression of *CsWRKY76* in sweet orange induced a concomitant rise in H_2_O_2_ levels and upregulated gene expression in the phenylpropanoid pathway. It was inferred from these results that a specific mechanism inhibits *CsWRKY76* expression at 37°C, thereby suppressing the activation of downstream immunity associated with *CsWRKY76*. In a suitable environment for sweet orange cultivation, *Cs*WRKY76 further activated *CsPR4A*. This process serves as a key regulatory mechanism against CYMV infection (Figure 6).

In conclusion, this study is the first to discover and confirm that high temperature promotes CYMV accumulation in sweet oranges. This is partly because sweet oranges experience weakened immunity under high temperatures, while CYMV possesses heat tolerance. Furthermore, we reveal that the *Cs*WRKY76‐*Cs*PR4A module plays a crucial role in modulating citrus resistance to CYMV infection. We provide a potential molecular target for the development of CYMV‐resistant citrus varieties.

## 4 Experimental Procedures

### 4.1 Plant Materials and Growth Conditions

Seven‐year‐old CYMV‐virulent and healthy Madam Vinous sweet orange plants were stored in the greenhouse of the Citrus Research Institute, in Beibei, Chongqing, China, where the temperature was controlled at 24°C–26°C. The CYMV infection of these trees was facilitated through grafting, and subsequent confirmation of the infections was achieved through PCR. In this study, the bark on the lower segment of the main trunk (without branches) was termed the old bark. The first three leaves located near the base of the branches growing upward from the main trunk were designated as the old leaves. The three terminal leaves on the main branches of citrus trees were classified as young leaves, while the bark adjacent to these leaves was termed young bark. Root samples were randomly collected from primary roots exhibiting lateral roots. All samples were dissected using surgical knives or scissors. After sampling, the samples were briefly rinsed with double‐distilled water and immediately frozen in liquid nitrogen. The seeds extracted from ‘jincheng’ sweet orange were used for the VIGS assay. *N. benthamiana* plants were cultivated in a plant incubator at 25°C in a 16 h: 8 h light: dark cycle.

### 4.2 DNA and RNA Extraction

The total DNA was extracted with the FastPure Plant DNA Isolation Mini Kit (Vazyme). The primers used for the PCR assays are listed in Table S2. The total RNA was extracted with the RNAiso Plus (Total RNA extraction reagent; Takara). The concentration and purity of the total DNA and RNA were then determined using a NanoDrop 2000 spectrophotometer (Thermo Scientific). The synthesis of cDNA was performed according to the instructions provided in the user guide of the PrimeScript II 1st Strand cDNA Synthesis Kit (Takara).

### 4.3 Real‐Time qPCR Assay

The SYBR qPCR SuperMix Plus (Takara) was used for qPCR. The following thermal cycling parameters were set on the CFX96TM Real‐Time System (Bio‐Rad): an initial hold at 95°C for 1 min; followed by 45 cycles of 95°C for 20 s, 58°C for 20 s and 72°C for 30 s; followed by a melting curve programme at 65°C to 95°C, raised gradually by 0.5°C every 5 s. The primers used for the RT‐qPCR assays are listed in Table S2. For analysis, the 2^−ΔΔ*C*t^ method was used, and the *COX* was used as the internal reference gene (Figure S3). Three technical replicates were set for each gene, and two biological replicates were performed.

### 4.4 Temperature‐Driven Annual Distribution of CYMV in Sweet Orange

To investigate the annual variation of CYMV content, the CYMV‐infected Madam Vinous sweet orange plants were cultivated in an insect‐proof net room at the Citrus Research Institute in Beibei, Chongqing, China. The temperature difference between the interior and exterior of the insect‐proof net room remained nearly identical. Daily temperature data of Beibei were obtained from the China Meteorological Administration (https://weather.cma.cn/). Details of temperature data processing are provided in Figure S4.

To further explore the impact of temperature on CYMV accumulation, a controlled experiment was conducted using Madam Vinous sweet orange plants infected with CYMV. The plants were maintained in a plant incubator. Two temperature groups were established: the control group was kept at 25°C, and the elevated temperature group at 37°C. Both groups followed a 14‐h light/10‐h dark cycle, with relative humidity maintained at 75% throughout the experiment.

### 4.5 Transcriptome Sequencing Analysis

Six CYMV‐infected Madam Vinous sweet orange plants were selected from section 4.3; three were maintained at 25°C (plants CY‐1, CY‐2 and CY‐3) and three at 37°C (CY‐4, CY‐5 and CY‐6) for 1 month. Subsequently, three randomly collected old leaves per plant were mixed and sent to Novogene Co. Ltd. (Beijing, China) for RNA‐Seq. The original sequenced data were filtered using Bowtie v. 2 (Langmead and Salzberg 2012). Clean reads were mapped to the reference genome of sweet orange (Xu et al. 2013) by HISAT v. 2.0.5 (Kim et al. 2015). The level of transcription of the genes in each sample was estimated by featureCounts software (Liao et al. 2014). DEGs were identified with edgeR with a filter threshold of FDR < 0.05 and log_2_foldchange > |1|. Gene function was annotated based on the GO database (https://www.geneontology.org) or the KEGG database (https://www.kegg.jp). The GO and KEGG enrichment analyses were calculated using Enrichr (Kuleshov et al. 2016).

### 4.6 Gene Cloning and Bioinformatics Analysis

The CDS of *CsWRKY76* and *CsPR4A* were cloned from Madam Vinous sweet orange via RT‐PCR. The relevant primers are shown in Table S2. The prediction of the binding of the *Cs*WRKY76 transcription factor with the *CsPR4A* promoter DNA fragment is based on Alphafold3 (http://alphafoldserver.com). The analysis of the protein–DNA interaction was conducted using the PDBePISA tool. The PyMOL v. 2.5.4 software (http://pymol.org) was used to define the interaction specifics, thereby generating a three‐dimensional interaction representation. The SGN VIGS Tool (https://vigs.solgenomics.net/) was used to design gene silencing fragments.

### 4.7 Subcellular Localisation Analysis

The CDS of *CsWRKY76* and *CsPR4A* (both without the termination codon) were cloned into the pCV‐GFP vector (Du et al. 2023), generating two fusion proteins: *Cs*WRKY76‐GFP and *Cs*PR4A‐GFP. The relevant primers are shown in Table S2. An empty pCV‐GFP vector was used as a control. The vector s were then individually transformed into *Agrobacterium tumefaciens* GV3101 (Weidi Biotechnology). The *A. tumefaciens* harbouring vector constructs were used to agroinfiltrate 4‐week‐old *N. benthamiana* plants as previously described (Tian et al. 2025). Three days after this process, at a temperature of 25°C, the presence of fluorescence signals in the transformed leaf tissue was observed by using an inverted fluorescence microscope (IX‐73; Olympus). The DAPI fluorescence excitation light wavelength was 405 nm and the GFP was 488 nm.

### 4.8 VIGS Assay

The citrus leaf blotch virus (CLBV)‐based system was used to silence the examined genes in Jincheng sweet orange plants (Wang et al. 2024). Partial CDS fragments of *CsWRKY76* (28–328 bp) and *CsPR4A* (1–300 bp) were amplified and inserted into the pCLBV201 vector (Wang et al. 2024). The relevant primers are shown in Table S2. The vectors were separately transformed into *A. tumefaciens* GV3101. The agrobacteria were cultivated in 5 mL LB medium with the appropriate antibiotics to an OD at 600 nm of 0.8–1.2. Subsequently, 2 mL of the bacterial culture from the previous step was inoculated into 200 mL of fresh LB medium. The bacterial solution was then transferred into a shaking table at 220 rpm at 28°C and agitated overnight to achieve an OD_600_ = 0.8. Centrifugation of the propagation solution was then conducted at 4°C and 6000 rpm for a duration of 5–10 min. The pelleted bacteria were then diluted to an OD_600_ of 0.6–0.8 using the bacterial suspensions, after which the samples were left undisturbed in the dark for 3 h. Subsequently, approximately 30 tissue‐cultured seedlings of Jincheng sweet orange were vacuum infiltrated for 60 s at 0.1 MPa as described (Wang et al. 2024). Any excess bacterial suspension was then removed from the surface of the seedlings using filter paper. The seedlings were then transplanted into Murashige and Skoog liquid medium, followed by a 2‐h treatment in darkness. Finally, the seedlings were transplanted into soil. The plants were then cultivated in a light incubator, and plant transformation was confirmed through PCR.

### 4.9 Analysis of Hairy Root Transformation Assay

The root‐mediated genetic transformation method was performed in accordance with Xiao et al. (2023). In order to construct the overexpression vector, the full‐length CDS of *CsWRKY76* and *CsPR4A* was cloned into the pLGN‐OE vector (Yao et al. 2020) containing a β‐glucuronidase (*GUS*) gene. An empty pLGN‐OE vector was used as a control. Partial CDS fragments of *CsWRKY76* (28–328 bp) and *CsPR4A* (1–300 bp) were amplified and inserted into the pLGN‐RNAi vector (Yao et al. 2020) containing a *GUS* gene to construct the interference vectors. The relevant primers are shown in Table S2. The vectors were separately transformed into *Agrobacterium rhizogenes* K599. The agrobacteria were cultivated in 5 mL LB medium with the appropriate antibiotics to an OD_600_ of 0.6–0.8. Subsequently, 2 mL of the bacterial culture from the previous step was inoculated into 200 mL of fresh LB medium. The bacterial solution was then transferred into a shaking table at 200 rpm at 28°C and agitated overnight to achieve an OD_600_ = 0.6 (Xiao et al. 2023). Centrifugation of the propagation solution was then conducted at 24°C and 5000 rpm for 5 min. The pelleted bacteria were then diluted to an OD_600_ of 0.4–0.6 using the bacterial suspensions, after which the samples were left undisturbed in the dark for a period of 2–4 h (Xiao et al. 2023). Subsequently, tender Madam Vinous sweet orange branches (dark green) were collected from the greenhouse, cut into 10–12 cm segments, and their lower ends were immersed in the bacterial suspension. The segments were then vacuum‐infiltrated for 30 min at 0.1 MPa. Finally, the branch segments were cultivated in a high‐humidity medium (a 3:1 volume mixture of soil and sand) in pots measuring 25.5 × 26.5 × 30.0 cm. The segments were grown at 25°C with a 16‐h/8‐h day/night photoperiod (Xiao et al. 2023). The histochemical assay of GUS activity was then carried out using transient expression in Madam Vinous sweet orange according to the manufacturer's protocol in a GUS staining kit (SL7160; Coolaber). The levels of H_2_O_2_ were determined using a hydrogen peroxide measuring kit (Solarbio).

### 4.10 Y1H Assay

The *CsPR4A* promoter sequence was cloned and inserted into the pAbAi vector (Fu et al. 2024), which was subsequently digested with BstBI (NEB). The linearised plasmid was subsequently transformed into the Y1H Gold yeast cells as bait. The prey plasmid pGADT7‐*Cs*WRKY76 was then transformed into the bait strain. The pAbAi‐p53 and pGADT7‐p53 vectors were transformed into the yeast strains as positive controls. The relevant primers are shown in Table S2. The pAbAi‐*proCsPR4A* and empty pGADT7 vectors were transformed into the yeast strains as the negative control. Single colonies were selected and cultivated on SD/−Leu medium with the appropriate cocnentraion of aureobasidin A (AbA) to confirm positive interactions.

### 4.11 Dual‐Luciferase Assay

The CDS of *CsWRKY76* was inserted into the pGreenII 62‐SK vector (Zhang et al. 2022) as the effector plasmid, and the promoter sequence of *CsPR4A* was inserted into the pGreenII 0800‐LUC vector (Zhang et al. 2022) as the reporter plasmid. The relevant primers are shown in Table S2. *A. tumefaciens* GV3101 was transformed by the freeze–thaw method, and PCR was used to confirm the positive clones (pGreenII 62‐SK‐*Cs*WRKY76 + pGreenII 0800‐LUC‐*proCsPR4A*). The following combinations were used: pGreenII 62‐SK + pGreenII 0800‐LUC *proCsPR4A*, pGreenII 62‐SK‐*Cs*WRKY76 + pGreenII 0800‐LUC and pGreenII 62‐SK + pGreenII 0800‐LUC. Subsequently, the suspensions were injected into the leaves of *N. benthamiana* with a volume ratio of the effector plasmid to the reporter plasmid of 9:1. After a 3‐day period, the *N. benthamiana* leaves were subjected to further analysis in accordance with the instructions provided in the dual luciferase reporter gene assay kit manual (Yeasen). The luciferase substrate, D‐luciferase potassium salt, was applied to the abaxial side of the injected *N. benthamiana* leaves at a concentration of 0.3 mg/mL. Subsequently, the leaves were subjected to a 20‐min period of darkness and imaged using the Image of Plant Living Imaging System (CCD) (Lumazone Pylon 2048B imaging system).

### 4.12 Statistical Analysis

The statistical analyses and mapping software were performed using GraphPad Prism software v. 10.3.0 (https://www.graphpad‐prism.cn/). For pairwise comparisons, significance analysis was calculated by Student's *t* test. For the purpose of conducting multiple‐group comparisons, significance analysis was calculated by one‐ or two‐way ANOVA. Asterisks are used to indicate significant differences (ns *p* ≥ 0.05, *p < 0.05, **p < 0.01, ***p < 0.001, ****p < 0.0001).

## Author Contributions

X‐.B.T., X.W. and Z.S. were involved in conceptualization. X‐.B.T., X.W., J.Z., J.L. and Y.L. were involved in methodology. X‐.B.T. and X.W. were involved in software. X‐.B.T., X.W. and Z.S. were involved in validation. X‐.B.T. and X.W. were involved in formal analysis. X‐.B.T., X.W. and Z.S. were involved in investigation. X‐.B.T. was involved in writing – original draft preparation. X‐.B.T. and Z.S. were involved in writing – review and editing. Z.S. was involved in funding acquisition. All authors have read and agreed to the published version of the manuscript.

## Conflicts of Interest

The authors declare no conflicts of interest.

## Supporting information


**Figure S1:** The relative expression of (a) *CsWRKY76* and (b) *CsPR4A* in the silenced jincheng sweet oranges.

**Figure S2:** (a) The relative expression of *CsWRKY76* in OE‐*CsWRKY76* Madam Vinous sweet orange hairy roots. (b) The GUS staining results of OE‐*CsWRKY76* Madam Vinous sweet orange hairy roots. (c) PCR‐diagnostic image of OE‐*CsWRKY76* Madam Vinous sweet orange hairy roots. (d) The relative expression of *CsPR4A* in OE‐*CsPR4A* Madam Vinous sweet orange hairy roots. (e) The GUS staining results of OE‐*CsPR4A* Madam Vinous sweet orange hairy roots. (f) PCR‐diagnostic image of OE‐*CsPR4A* Madam Vinous sweet orange hairy roots. (g) The relative expression of *CsWRKY76* in RNAi‐*CsWRKY76* Madam Vinous sweet orange hairy roots. (h) The GUS staining results of RNAi‐*CsWRKY76* Madam Vinous sweet orange hairy roots. (i) PCR‐diagnostic image of RNAi‐*CsWRKY76* Madam Vinous sweet orange hairy roots. (j) The relative expression of *CsPR4A* in RNAi‐*CsPR4A* Madam Vinous sweet orange hairy roots. (k) The GUS staining results of RNAi‐*CsPR4A* Madam Vinous sweet orange hairy roots. (l) PCR‐diagnostic image of RNAi‐*CsPR4A* Madam Vinous sweet orange hairy roots. M: DNA marker DL1500. Lane, H_2_O: negative control. +: positive control.

**Figure S3:** The cycle threshold value of *CsCOX* in sweet orange leaves at 25°C and 37°C was determined by reverse transcription‐quantitative PCR assay.

**Figure S4:** The average high temperature was calculated by summing the daily maximum temperatures recorded during the statistical period and dividing the total by the number of days. The average low temperature was similarly calculated using daily minimum temperatures. The extreme high temperature denotes the highest temperature observed throughout the period, whereas the extreme low temperature refers to the lowest temperature recorded during the same interval.

**Table S1:** mpp70161‐sup‐0005‐TableS1.xlsx.

**Table S2:** mpp70161‐sup‐0006‐TableS2.xlsx.

## Data Availability

The data that support the findings of this study are available from the corresponding author upon reasonable request.
