# Supplementary figures and images for: High Temperature Promoted the Accumulation of Citrus Yellow Mosaic Virus in Citrus sinensis via Weakening the Immune Function of the CsWRKY76‐ CsPR4A Modules
Source: Mol Plant Pathol. 2025 Oct 5;26(10):e70161. doi: 10.1111/mpp.70161 (PMC12497541; doi:10.1111/mpp.70161)

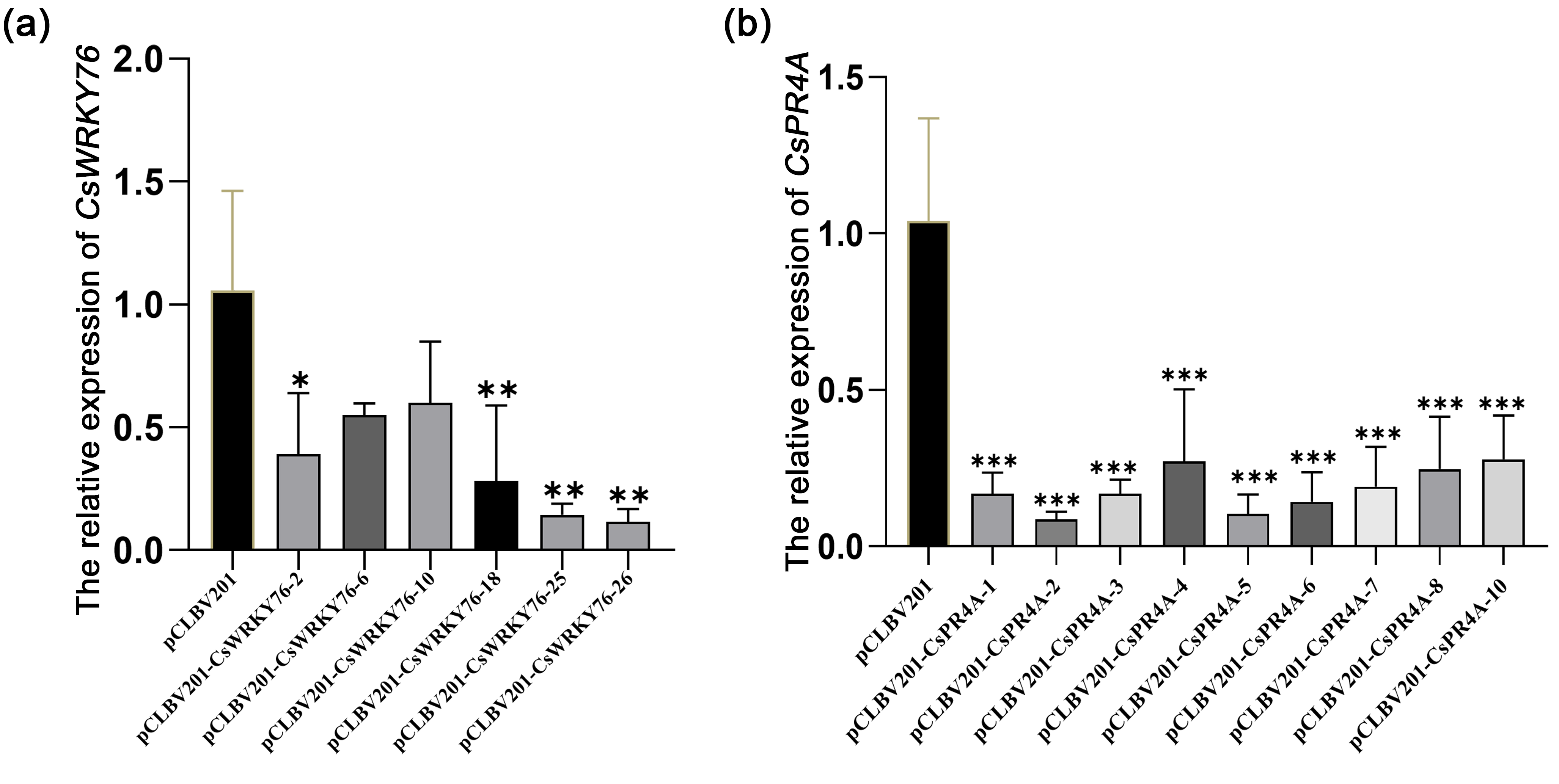

Supplement: Supplementary file 1 — Figure S1: The relative expression of (a) CsWRKY76 and (b) CsPR4A in the silenced jincheng sweet oranges. [file MPP-26-e70161-s002.tif]

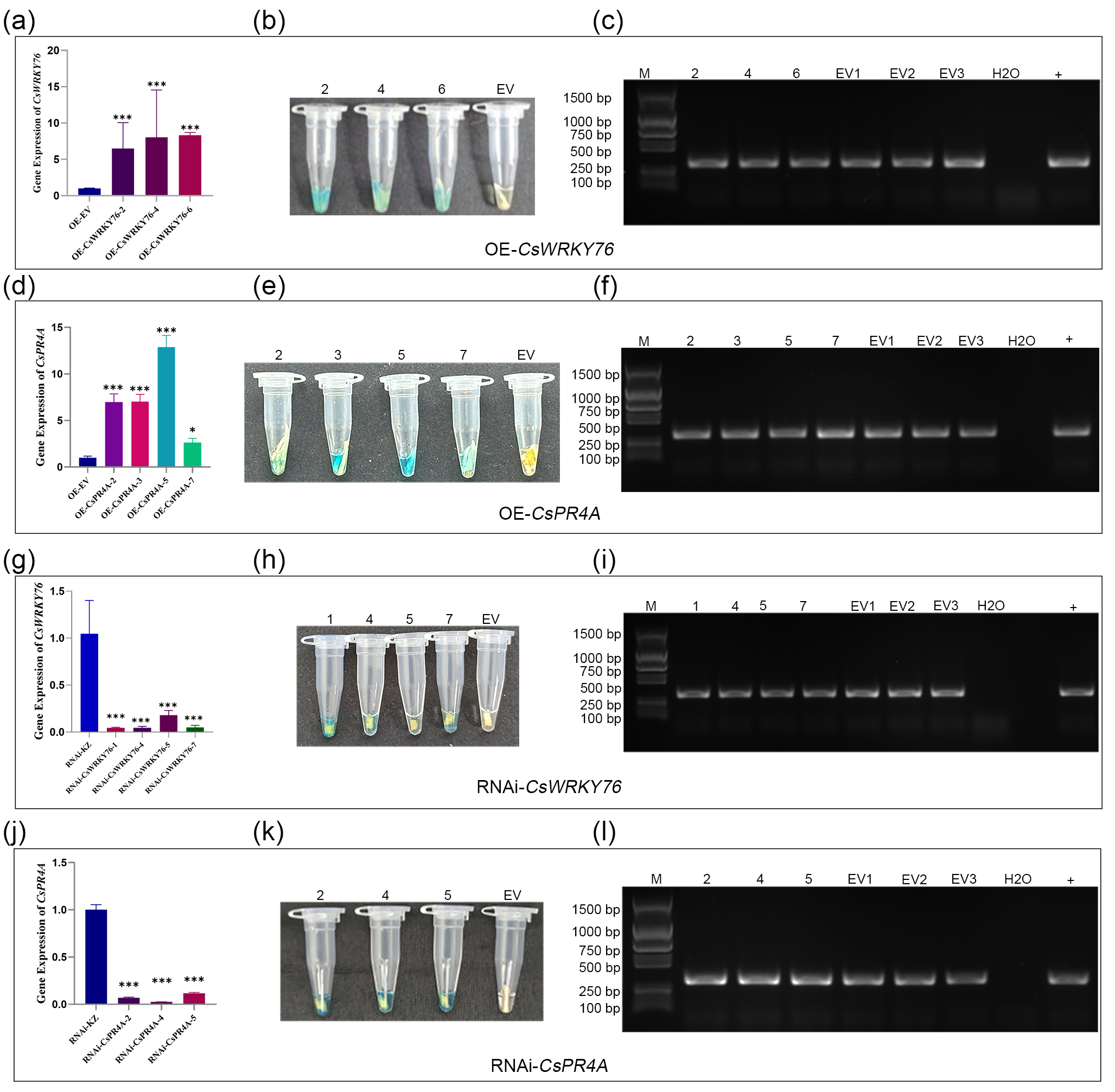

Supplement: Supplementary file 2 — Figure S2: (a) The relative expression of CsWRKY76 in OE‐CsWRKY76 Madam Vinous sweet orange hairy roots. (b) The GUS staining results of OE‐CsWRKY76 Madam Vinous sweet orange hairy roots. (c) PCR‐diagnostic image of OE‐CsWRKY76 Madam Vinous sweet orange hairy roots. (d) The relative expression of CsPR4A in OE‐CsPR4A Madam Vinous sweet orange hairy roots. (e) The GUS staining results of OE‐CsPR4A Madam Vinous sweet orange hairy roots. (f) PCR‐diagnostic image of OE‐CsPR4A Madam Vinous sweet orange hairy roots. (g) The relative expression of CsWRKY76 in RNAi‐CsWRKY76 Madam Vinous sweet orange hairy roots. (h) The GUS staining results of RNAi‐CsWRKY76 Madam Vinous sweet orange hairy roots. (i) PCR‐diagnostic image of RNAi‐CsWRKY76 Madam Vinous sweet orange hairy roots. (j) The relative expression of CsPR4A in RNAi‐CsPR4A Madam Vinous sweet orange hairy roots. (k) The GUS staining results of RNAi‐CsPR4A Madam Vinous sweet orange hairy roots. (l) PCR‐diagnostic image of RNAi‐CsPR4A Madam Vinous sweet orange hairy roots. M: DNA marker DL1500. Lane, H2O: negative control. +: positive control. [file MPP-26-e70161-s003.tif]

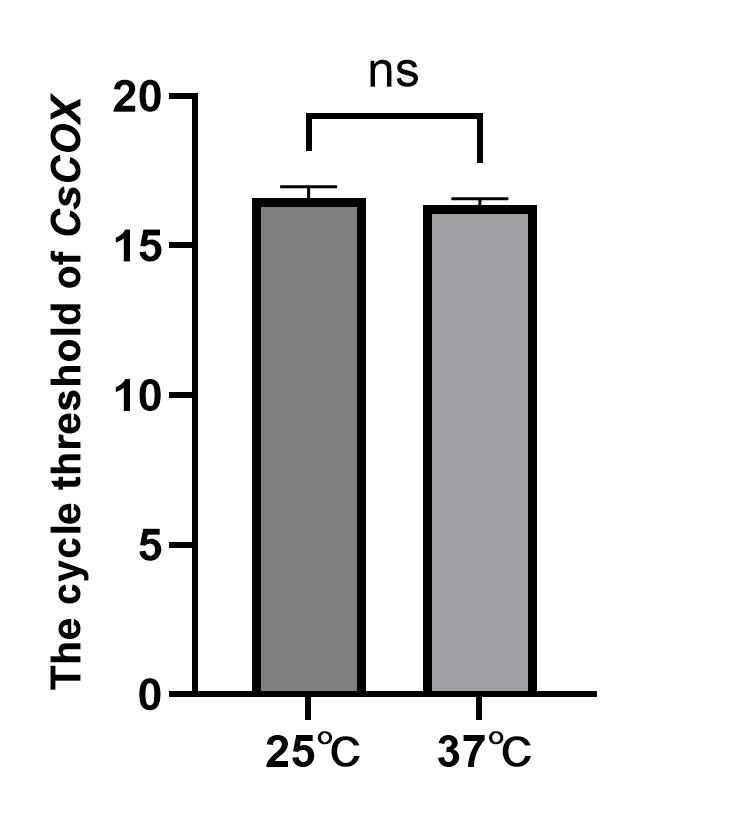

Supplement: Supplementary file 3 — Figure S3: The cycle threshold value of CsCOX in sweet orange leaves at 25°C and 37°C was determined by reverse transcription‐quantitative PCR assay. [file MPP-26-e70161-s006.tif]

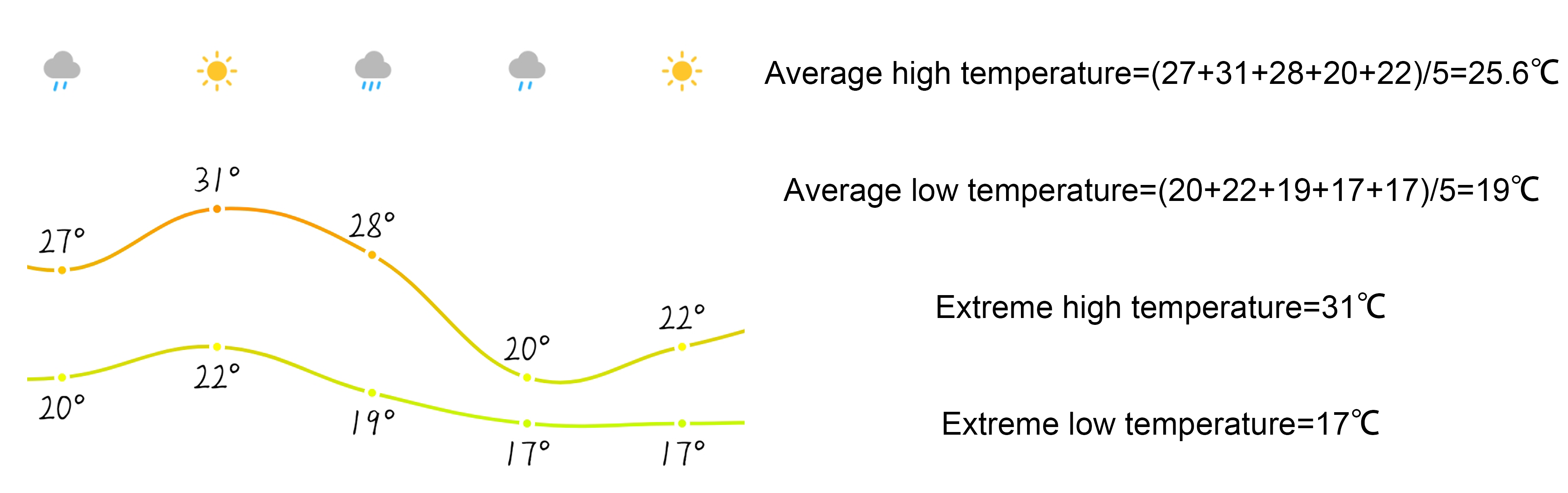

Supplement: Supplementary file 4 — Figure S4: The average high temperature was calculated by summing the daily maximum temperatures recorded during the statistical period and dividing the total by the number of days. The average low temperature was similarly calculated using daily minimum temperatures. The extreme high temperature denotes the highest temperature observed throughout the period, whereas the extreme low temperature refers to the lowest temperature recorded during the same interval. [file MPP-26-e70161-s004.tif]
